# Stromal Modulators of TGF-β in Cancer

**DOI:** 10.3390/jcm6010007

**Published:** 2017-01-06

**Authors:** Brunella Costanza, Ijeoma Adaku Umelo, Justine Bellier, Vincent Castronovo, Andrei Turtoi

**Affiliations:** 1 Metastasis Research Laboratory, GIGA-Cancer, University of Liege, 4000 Liege, Belgium; brunellacostanza@gmail.com (B.C.); iumelo@ulg.ac.be (I.A.U.); justine.bellier@ulg.ac.be (J.B.); vcastronovo@ulg.ac.be (V.C.); 2 Institut de Recherche en Cancérologie de Montpellier (IRCM), INSERM U1194, Université Montpellier, Institut Régional du Cancer de Montpellier, 34298 Montpellier, France

**Keywords:** cancer-associated fibroblasts, proteases, proteoglycans, TGF-β, stroma

## Abstract

Transforming growth factor-β (TGF-β) is an intriguing cytokine exhibiting dual activities in malignant disease. It is an important mediator of cancer invasion, metastasis and angiogenesis, on the one hand, while it exhibits anti-tumor functions on the other hand. Elucidating the precise role of TGF-β in malignant development and progression requires a better understanding of the molecular mechanisms involved in its tumor suppressor to tumor promoter switch. One important aspect of TGF-β function is its interaction with proteins within the tumor microenvironment. Several stromal proteins have the natural ability to interact and modulate TGF-β function. Understanding the complex interplay between the TGF-β signaling network and these stromal proteins may provide greater insight into the development of novel therapeutic strategies that target the TGF-β axis. The present review highlights our present understanding of how stroma modulates TGF-β activity in human cancers.

## 1. Introduction

**Figure 1:**
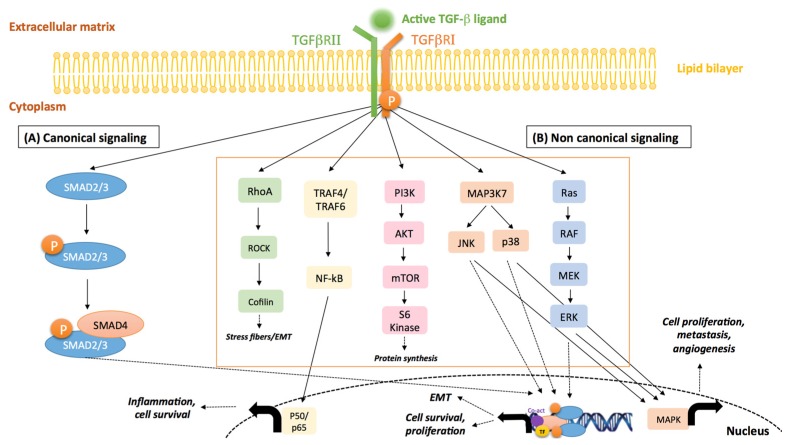
Canonical and non-canonical TGF-β signaling pathways. (**A**) In the canonical signaling pathway, biologically active TGF-β ligands bind to TGFβRII, which in turn activates TGFβRI. TGFβRI-regulated SMAD2/3 proteins are phosphorylated at their C-terminal serine residues and form complexes with SMAD4 (co-SMAD), initiating a number of biological processes through transcriptional regulation of target genes. (**B**) In the non-canonical signaling pathways, the TGF-β receptor complex transmits its signal through other factors, such as the mitogen-activated protein kinases (MAPKs), phosphatidylinositide 3-kinase (PI3K), TNF receptor-associated factor 4/6 (TRAF4/6) and Rho family of small GTPases. Activated MAPKs can exert transcriptional regulation either through direct interaction with the nuclear SMAD protein complex or via other downstream proteins. Moreover, activated JNK/p38/ERK act in concert with SMADs to regulate cellular apoptosis and proliferation, whereas they mediate metastasis, angiogenesis and cellular growth through other transcription factors, such as c-JUN and ATF. RhoA/ROCK can be activated by TGF-β to induce actin stress fiber formation during EMT via a non-transcriptional mechanism. TGF-β can activate PI3K and AKT by inducing a physical interaction between the PI3K p85 subunit and the receptor complex leading to translational responses via mTOR/S6kinase activation. TGF-β activation of the TRAF proteins can initiate nuclear factor-κB (NF-κB) signaling activity, leading to the inflammatory response among other processes. The arrows indicate activation/signaling direction of the respective pathway.

The transforming growth factor-β ligands (TGF-β1, TGF-β2 and TGF-β3) are members of a super family of secreted cytokines that regulate a variety of physiological cellular processes, including proliferation, differentiation, migration, survival and immunity [1,2,3]. In its active form, TGF-β signals to the nucleus mainly through its cognate receptors, TGF-β type I and type II receptors (TGFβRI and TGFβRII), which phosphorylate canonical SMAD2/3 downstream transducers (Figure 1) [4,5]. In addition, several factors, such as various mitogen-activated protein kinases (MAPKs; namely, the extracellular signal-regulated kinase (ERK), c-Jun N-terminal kinase (JNK)and p38 MAPK), phosphatidylinositide 3-kinase (PI3K)/AKT, Rho-like GTPases (Rho) and TNF receptor-associated factor 4/6 (TRAF 4/6), can be activated by TGF-β via non-canonical signaling cascades (Figure 1) [6]. Of note, emerging evidence has revealed that both TGF-β canonical and non-canonical signaling cascades can simultaneously occur through crosstalk of core pathway components and combined utilization of SMAD/non-SMAD transcription factors [7] (Figure 1). A number of excellent reviews have extensively covered TGF-β signal transduction [8,9,10,11]. 

Cancer cells use TGF-β in order to enhance their characteristic properties and features [2,12,13,14]. In the transition from a physiological to pathological phenotype, TGF-β can induce intrinsic dichotomous effects, which reflect both its tumor suppressive and tumor promoting function. Although this dual role of TGF-β in cancer is poorly understood [15,16], it is known that the stage of progression and cellular context are key factors. In epithelial cells and during tumor initiation, TGF-β acts as a tumor suppressor by inhibiting the growth of malignant cells via canonical SMAD2/3 signaling activity [8,17]. Evidence also suggests that the pro-apoptotic effects of TGF-β contribute to its observed cytostatic features during early tumor formation. TGF-β-induced apoptosis has been observed to occur with SMAD [18], JNK [19] and p38 [20] signaling activity in neoplastic epithelium. In later stages of malignant progression, however, cells can induce the loss of the tumor suppressive activities of TGF-β signaling by acquiring mutations or alterations in canonical target genes. This switch in response to TGF-β by tumor cells is accompanied by epithelial-to-mesenchymal transition (EMT), as well as the enhancement of a number of cancer cell hallmarks, including angiogenesis, invasion and metastasis [8,21]. Accordingly, pre-clinical and clinical observations support the prevailing hypothesis that TGF-β exhibits two opposing effects in tumor control and progression. Experimental evidence suggests that the TGF-β signaling network can indeed switch from tumor suppressor to tumor promoter, especially in the presence of oncogenic events and epigenetic perturbations [17]. Recently, PEAK1 (a novel non-receptor tyrosine kinase), highly expressed in invasive breast cancer, has been described as relevant for switching TGF-β from a tumor suppressor to a tumor-promoting factor [22]. Agajanial et al. provide preclinical evidence that PEAK1 potentiates TGF-β-mediated proliferation and tumor progression in a fibronectin-dependent fashion. In the presence of fibronectin, PEAK1 causes a switch from canonical TGF-β SMAD2/3 signaling to non-canonical Src and MAPK signaling [22]. In line with this, other pre-clinical findings support the idea that a balance shift between TGF-β-induced SMAD-dependent and -independent signaling activity is the underlying basis for TGF-β-induced tumor progression [7]. To this already complex dual role, an additional level of complexity is added by stromal-derived enhancer and suppressor molecules that can directly modulate TGF-β activity [23]. Tumor stroma, an abundant source of TGF-β, comprises a large array of epithelial, fibroblast, endothelial and inflammatory cell populations that coordinate together to regulate tumor growth and progression [24]. Of these, cancer-associated fibroblasts (CAFs) are a unique cellular subset due to their predominantly complex interaction with cancer cells. This complexity is further reflected in the dual function that CAFs can exhibit during the course of tumor progression, in either maintaining or inhibiting a variety of cancer cell-related processes [25]. Much of this interaction is transmitted through CAF-mediated TGF-β signaling, where CAFs themselves are significant contributors of overall levels of TGF-β in the tumor [26,27]. It is worth noting, however, that the concentration of TGF-β in the tumor or the production of TGF-β by cancer cells or CAFs does not directly translate to the bioavailability of this cytokine and, hence, its activity. Indeed, TGF-β is secreted in the microenvironment as an inactive form called small latent complex (SLC) [28]. Latency occurs due to the non-covalent interaction between the functional TGF-β ligand and its cleaved pro-domain, latency-associated peptide (LAP). In the extracellular matrix (ECM), TGF-β activators release the active cytokine from its interaction with LAP through proteolytic cleavage or structural modifications, such as deglycosylation [2,28,29]. To date, the identity and function of stromal factors that regulate the conversion of latent TGF-β to active TGF-β [30] or inhibit TGF-β activity by binding to the cytokine or its cognate receptors have been constantly emerging. These factors are critical in understanding TGF-β function as it relates to tumor formation and progression and, as such, represent the main subject of the present review. 

## 2. Cancer Cell/Stroma Crosstalk and TGF-β Activity

Clinical observations have demonstrated that elevated expression of stromal-derived TGF-β is associated with poor prognosis and locally-advanced disease in breast cancer [31], colorectal cancer [26,27,32] and prostate cancer [33]. Stromal-derived TGF-β can activate resident stromal cells via autocrine signaling, giving rise to tumor-promoting stromal cells, such as CAFs and myofibroblasts. These activated stromal cells can in turn induce the upregulation of paracrine factors, such as HGF [34], CXCL1 and CXCL16 [35], which promote EMT and the induction of epithelial invasion in adjacent epithelial cells [36]. In addition, other studies have provided supportive evidence that TGF-β secreted by epithelial cancer cells exerts a paracrine influence on stromal cells resulting in increased production of ECM and enhanced tumor proliferation [37,38]. Accordingly, given this pleiotropic role of TGF-β in malignant progression, we examine in this section its impact on some key cancer hallmarks, focusing in particular on cancer cell-stroma cell interactions.

### 2.1. Metastasis

**Figure 2:**
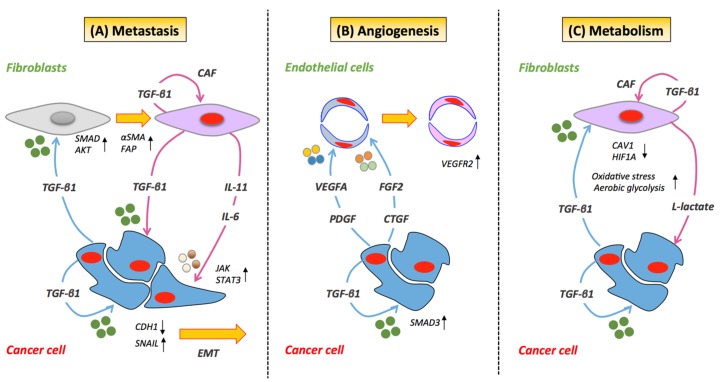
TGF-β-mediated cancer cell/stromal cell crosstalk. (**A**) TGF-β can activate resident stromal cells giving rise to cancer-associated fibroblasts (CAFs). In cancer cells, TGF-β promotes the transcription of SNAIL, the functional loss of E-cadherin, the acquisition of an EMT phenotype and the recruitment of SMAD/AKT signaling proteins. The process of metastasis is further supported by activated CAFs through secretion of IL-11 or IL-6, which further promotes STAT3 signaling in cancer cells. (**B**) TGF-β can trigger angiogenesis in endothelial cells through activation of VEGFR2 by VEGF. The TGF-β-mediated angiogenic effect on cancer cells is regulated by TGFβRII/SMAD3-dependent upregulation of fibroblast growth factor-2 (FGF2) expression and release in the stroma. (**C**) Cancer cells via the induction of aberrant TGF-β signaling can induce the down-regulation of CAV1 in adjacent fibroblasts leading to a CAF phenotype. The loss of CAV1 has been observed to lead to an increase in oxidative stress, activation of HIF-1α and the induction of aerobic glycolysis. Under these conditions, CAF have been reported to produce and secrete lactate, which is used as fuel by cancer cells. Blue arrows indicate proteins secreted by cancer cells. Magenta arrows indicate proteins secreted by stromal cells. Black arrows indicate overexpression (upward pointing) and down-regulation (downward pointing) of target proteins.

TGF-β plays an important role in cancer metastasis [8,26,39], essentially by stimulating the invasive and metastatic potential of epithelial cells. This occurs in part through its induction of classical EMT mechanisms and by driving the intravasation and extravasation of malignant cells to distal sites. TGF-β-induced EMT has been reported to integrate both SMAD- and non-SMAD-dependent signaling elements, requiring crosstalk between PI3K/AKT and SMAD signaling proteins (Figure 1) [40]. Importantly, collective data have described the transcription factor SNAIL as a key mediator in TGF-β-induced EMT [40,41,42]. TGF-β1-induced activation of SNAIL has also been reported to promote mesenchymal transition in colon cancer cells [43] breast cancer cells [44], lung cancer cells [45] and endothelial cells [46]. TGF-β exerts its influence on SNAIL in a two-step process, by increasing its transcription and repressing E-cadherin through the recruitment of SMAD proteins [40]. Inhibition of SNAIL with anti-sense oligonucleotides blocks TGF-β-induced EMT and AKT phosphorylation, suggesting that SNAIL participates in TGF-β-induced EMT by acting upstream of AKT [40]. In lung cancer, the functional loss of E-cadherin and the acquisition of an EMT phenotype in epithelial cells have been reported to occur through TGF-β1-induced transcription of SNAIL via collaboration of the IL-6/STAT3 signaling pathway (Figure 2A) [45]. Other investigations have demonstrated that genes upregulated by stromal-derived TGF-β (e.g., *JAG1*, *CTGF* and *TNC*) are predictors of recurrent and metastatic disease in colorectal cancer [26]. The investigators also reveal that the initiation of metastasis by TGF-β-induced stromal cells is dependent on GP130/STAT3 signaling in cancer cells via paracrine secretion of IL-11 (Figure 2A). This tumor-stroma crosstalk consequently provides a survival advantage to metastatic cells and correlates with a high risk of treatment failure and relapse in the metastatic setting. In addition, in xenografts of breast cancer, the presence of TGF-β has been shown to promote metastasis by enhancing the motility of stromal cells expressing α smooth muscle actin (α-SMA) and vimentin [47].

### 2.2. Angiogenesis

In later stages of cancer development, TGF-β potently stimulates angiogenesis, mainly through its canonical signaling cascades [48]. In addition, TGF-β knock-out mice are not viable and display a phenotype defective in vasculogenesis and angiogenesis [48,49]. The role of TGF-β in the angiogenesis is multimodal. For example, the TGF-β-mediated angiogenic effect on xenografts of prostate cancer is regulated by TGFBRII/SMAD3-dependent upregulation of fibroblast growth factor-2 (FGF2) expression and release in prostate stroma (Figure 2B) [50]. In addition to FGFD2, TGF-β affects the expression of other stromal-derived angiogenic factors, including vascular endothelial growth factor (VEGF) [51,52], connective tissue growth factor (CTGF) [53] and platelet-derived growth factor (PDGF) [54]. Interestingly, TGF-β-induced endothelial cell apoptosis has been reported to trigger angiogenesis through paracrine and autocrine activation of VEGFR2 by VEGF (Figure 2B) [55]. On the other hand, other lines of pre-clinical research have presented TGF-β as an anti-angiogenic factor. Recent evidence in experimental models of CRC has revealed that TGF-β-mediated signaling under hypoxic stress conditions promotes decreased VEGFA expression, thus reducing VEGFA-induced angiogenesis (Figure 2B). The investigation also indicates that TGF-β regulates VEGFA at the post-transcriptional level by decreasing VEGFA protein stability through ubiquitination and degradation [56]. While the specific mechanism of TGF-β-mediated control of pro-angiogenic and anti-angiogenic processes needs to be fully explored, preclinical evidence suggests that it may be dependent on the concentration of TGF-β in the endothelium and distinct SMAD signaling cascades [48,57,58]. In endothelial cells, TGF-β-mediated inhibition of angiogenesis occurs through the TGFβRI/ SMAD2/3 signaling cascade [58,59], while its stimulatory influence on angiogenesis arises via plasminogen-dependent activation of TGFβRI/ SMAD1/5 [58].

### 2.3. Metabolism

TGF-β promotes metabolic alterations in the tumor microenvironment via “metabolic reprogramming” of CAFs through aberrant TGF-β signaling and loss of stromal caveolin-1 (CAV1) [37]. Moreover, pre-clinical investigations have demonstrated that epithelial cancer cells can induce the down-regulation of CAV1 in adjacent fibroblasts, leading to a specific, tumor-supportive, CAF phenotype (Figure 2C). While the precise mechanism is still not well understood, loss of CAV1 has been observed to lead to increased oxidative stress, activation of HIF1α and the induction of aerobic glycolysis/the Warburg effect in the tumor microenvironment [60,61]. Pre-clinical evidence has also demonstrated that the loss of CAV1 regulates stromal TGF-β via induction of aberrant TGF-β signaling [62]. Similarly, overexpression of TGF-β in stromal cells alters the CAF phenotype and promotes tumorigenesis by causing a shift towards catabolic metabolism [37]. These metabolic alterations in CAFs can result in an increased production of high-energy metabolites, such as L-lactate and ketone bodies [37,62], potentially further fueling the anabolic growth of adjacent cancer cells [63].Additional experimental evidence has also shown that TGF-β-induced influence of activated CAFs on cancer cells can also enhance their mitochondrial activity [37]. 

## 3. Stromal Activators of TGF-β in Cancer

It is well documented that the interaction between the latent TGF-β complex, TGF-β activators and components of the ECM exerts a regulatory impact on active TGF-β levels [64,65,66]. Several stromal-derived factors, including proteases, integrins and reactive oxygen species (ROS), have been implicated in the activation of latent TGF-β. In this section, we examine known stromal activators of TGF-β, although many of the described molecules are not exclusively stromal-derived, but also produced by cancer cells themselves.

### 3.1. Matrix Metalloproteinases

The matrix metalloproteinases (MMPs) are a multi-gene family of zinc-dependent proteases produced by malignant epithelial and adjacent stromal cell populations. These enzymes are involved in the proteolysis of ECM components and actively participate in several steps of malignant progression, such as tissue remodeling and cell migration. The role of MMPs in tumor progression has been summarized in a number of recent reviews [67,68,69,70].

**Figure 3:**
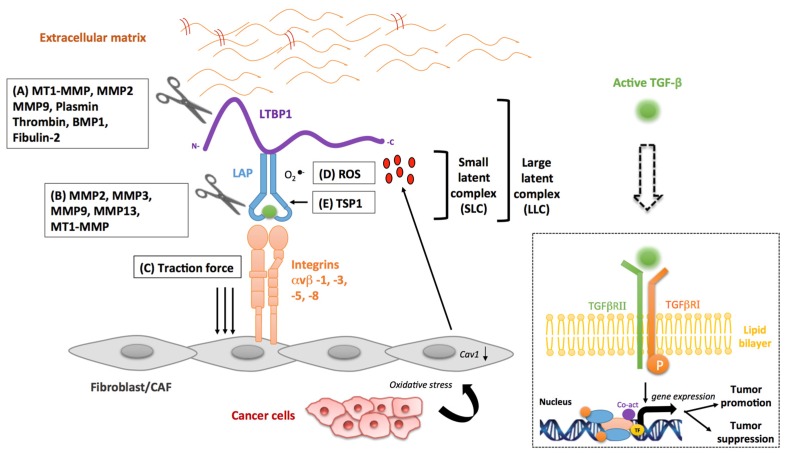
Stromal activators of TGF-β in the tumor microenvironment. (**A**) MT1-MMP, MMP2 and MMP9 proteolytically cleave latent transforming growth factor-β binding protein (LTBP), thereby releasing latent TGF-β from the extracellular matrix. Plasmin, thrombin, BMP1 and fibulin-2 also activate TGF-β through cleavage or interaction with LTBP1. (**B**) MMP2, MMP3, MMP9 and MMP13 activate latent TGF-β via proteolytic cleavage of the latency-associated peptide (LAP), while integrins expressed on fibroblasts (αvβ3, αvβ5 and αvβ8) bind to the large latent complex (LLC) and activate latent TGF-β through MT1-MMP-dependent cleavage of LAP. (**C**) Integrins αvβ-1 and 5 bind to the LLC and induce conformational changes in the latent complex via contractile action from activated fibroblasts. (**D**) ROS produced by activated fibroblasts via the induction of oxidative stress from adjacent cancer cells can lead to the oxidation of the LAP domain and induce allosteric changes that release mature TGF-β from LAP. The loss of CAV1 expression in activated fibroblasts is also associated with enhanced oxidative stress and increased production of ROS. (**E**) Thrombospondin-1 (TSP-1) directly interacts with the LAP domain, inducing conformational rearrangement of LAP and altering the interaction of LAP with the mature domain of TGF-β. The mature (active) form of TGF-β can then bind to its cognate receptor and exert its tumor promoting and tumor suppressive properties. Dashed arrow indicates recruitment of the mature TGF-β protein to its cognate receptor.

Besides their established pro-tumorigenic function, a number of MMPs, such as membrane type 1 matrix metalloproteinase (MT1-MMP), MMP2, MMP3, MMP9 and MMP13, have been described as key elements in the stromal activation of latent TGF-β (Figure 3A) [71,72,73,74,75]. Studies have demonstrated that MT1-MMP, MMP2 and MMP9 release latent TGF-β1 from the ECM by proteolytic cleavage of the large latent TGF-β1 binding protein-1 (LTBP1) [76]. Moreover, a preclinical investigation by Tatti et al. has demonstrated the particular importance of the MMPs in LTBP1 cleavage [77]. The authors have found that shRNA-mediated knockdown of MMP expression or potent pharmacological inhibition of MMP activity prevents the release of LTBP1 from the ECM [77], thus limiting the availability of soluble TGF-β. In addition, it has recently been shown that the LH3 receptor acts as a docking site in recruiting MMP9 to the surface of fibroblasts, which can trigger TGF-β activation and myofibroblast differentiation [78]. Myofibroblasts, in turn, enhance tumor progression by remodeling the stroma. In comparison, MMP9 localized on the surface of cancer cells can also activate latent TGF-β, albeit in a CD44-dependent manner [75].CD44 provides a cell surface docking receptor for proteolytically-activeMMP9, resulting in TGF-β-induced myofibroblasts formation [75]. Proteolytic cleavage of mature TGF-β from LAP by MMP2, MMP3, MMP9 and MMP13 has also been reported to release active TGF-β in the ECM (Figure 3B) [66]. Maeda et al. have proposed a model where MMP3-mediated activation of latent TGF-β1 consists of several steps, including proteolytic cleavage of several novel sites of the LTBP1 molecule and subsequent dissociation of the mature TGF-β protein from the LAP propeptide [73]. The authors also demonstrate that treatment with an anti-MMP3 antibody results in the dose-dependent decrease of active TGF-β1. It is important to note, however, that once activated, TGF-β can also regulate the secretion, expression and activation of all of the aforementioned MMPs, resulting in a bidirectional regulatory loop [64,67].

### 3.2. Integrins

The integrins are a family of cell surface adhesion molecules that are known to play an important role in cell-ECM interactions and fibrosis [79]. These molecules contribute to the stromal activation of latent TGF-β mainly by two non-mutually-exclusive mechanisms: (i) protease-dependent interaction of latent TGF-β and MMPs (Figure 3A,B) [80]; or (ii) induction of conformational changes in the latent TGF-β complex due to the generation of contractile forces (Figure 3C) [81].

The first integrin identified as an activator of TGF-β1 and TGF-β3 was αvβ6, an integrin solely expressed in epithelial cells [82]. However, in fibroblasts, integrin αvβ8 has been reported to mediate the activation of various TGF-β isoforms through binding with high affinity to the integrin binding motif (RGD) of the latent TGF-β LAP domain [74]. This coordinated binding leads to MT1-MMP-dependent release of active TGF-β at the cell surface [74]. Further to this, integrins can also contribute to TGF-β activation independent of MMP-mediated proteolysis. Indeed, a recent study by Sheppard and collaborators has demonstrated the putative role of fibroblast-derived αvβ1 in the activation of TGF-β1 in lung and liver fibrosis. The investigation shows that αvβ1 is able to directly bind the LAP domain of TGF-β1, which leads to the release of mature TGF-β1by contractile forces [83]. The experimental data also suggest that targeting αvβ1 with a specific inhibitor could be a potential therapeutic component in the treatment of lung and liver fibrosis [83]. Wipff et al. have also recently demonstrated that integrin αvβ5 can activate TGF-β1 on the surface of myofibroblasts, in the absence of proteases [81]. Mechanistically, the authors demonstrate that myofibroblast contraction can lead to this activation of latent TGF-β1. The cell traction forces that myofibroblasts transmit to the LAP domain generate allosteric changes in the latent complex and the subsequent release of the active form of TGF-β1 [81]. 

An additional line of evidence has also shown that integrins αvβ3 and αvβ5 can contribute to the establishment of an autocrine TGF-β loop through the activation of TGF-β1, in an MMP-independent fashion [84]. Integrins αvβ3 and αvβ5 are overexpressed in scleroderma fibroblasts compared to normal fibroblasts, where this altered expression increases the promoter activity of human collagen α-2 [84]. Systemic sclerosis is an immune-mediated, multi-system disorder characterized by microvasculature damage, circulating auto-antibodies and fibroblast activation. The findings also reveal that pre-treatment with anti-αvβ3 and anti-αvβ5 antibodies can reverse the myofibroblastic phenotype of scleroderma-derived fibroblasts, indicating that these integrins are crucial players in sclerosis pathogenesis [85]. A meta-analysis involving more than 7000 patients has estimated that sclerodermic patients have a 75% increased risk of developing cancers of the lung compared to the general population [86].

### 3.3. Reactive Oxygen Species

In cancer, the excessive production of reactive oxygen species (ROS) can induce chemical damage in proteins, lipids and DNA contributing to diverse tumorigenic effects [87]. While ROS are not a specific feature of stromal cells, cancer cells induce increased ROS production in CAFs. This in turn mediates CAF senescence and metabolic reprogramming. TGF-β has been implicated in a number of reactive oxygen-mediated tissue processes, particularly inflammation [88]. Importantly, radiation exposure, known to generate ROS, has also been demonstrated as a factor in TGF-β1 activation [89]. Barcellos-Hoff et al. have shown that irradiation of the latent TGF-β complex can significantly increase TGF-β activity. The authors further propose that ROS can oxidize specific amino acids in the LAP domain of the latent TGF-β complex, inducing conformational changes that release active TGF-β from the ECM (Figure 3D) [89]. 

As described earlier in this review, deregulated metabolism and oxidative stress in CAFs is associated with the loss of CAV1 expression [37]. This loss of CAV1 also correlates with an increase in ROS production, leading to enhanced TGF-β1 secretion and aberrant activation of the TGF-β signaling pathway [37]. In support of these observations, a number of independent studies has highlighted the direct role of CAV1 in the TGF-β signaling axis. For example, Ayala et al. have shown that silencing of CAV1 in prostatic fibroblasts results in increased TGF-β1 gene expression in tumor cells [90]. Similarly, other groups have established that inhibition of endogenous CAV1 in murine fibroblasts increases *TGFβRII* gene expression, whereas its sustained expression suppresses TGF-β-mediated cellular processes [91]. Recently, Jain et al. have demonstrated that mitochondrial-derived ROS plays an important role in regulating TGF-β in normal human lung fibroblasts [92]. Their results further reveal that the use of anti-oxidants targeting mitochondrial ROS significantly attenuates TGF-β gene expression, without affecting SMAD phosphorylation or nuclear translocation [92]. However, only the latent form of TGF-β1 seems to be sensitized by redox-mediated activation. Another investigation has determined that sensitivity to ROS activation depends on a conserved methionine residue in LAP-β1. The study has also demonstrated that altering this conserved residue with a site-specific mutation disrupts the sensitivity of LAP-β1 to ROS-mediated activation [93].

### 3.4. Other Stromal Activators of TGF-β

In addition to the MMPs, integrins and ROS, several other stromal-derived molecules are also known for their ability to activate TGF-β. Experimental evidence has revealed that the ECM protein, thrombospondin-1 (TSP-1), can activate latent TGF-β secreted by several cell types, including fibroblasts and mammary epithelial cells [94]. TSP1 can bind to both the LLC and the SLC forms of latent TGF-β [95]. Moreover, Ribeiro et al. have revealed that TSP1-mediated activation of latent TGF-β1 occurs by direct interaction of complementary residues in TSP1 and LAP, leading to the conformational rearrangement of LAP and the modulation of its interaction with the mature domain of TGF-β (Figure 3E) [96]. Interestingly, TSP1-null and TGF-β1-null mice demonstrate a phenotypic overlap with strikingly similar developmental abnormalities [97]. In addition, a co-operative mechanism for TGF-β activation, involving TSP-1 and plasmin, has been described. The mechanism was demonstrated in a bleomycin-dependent model of pulmonary fibrosis [98]. TSP-1 is, therefore, able to activate latent TGF-β through different mechanisms in different circumstances with beneficial or deleterious consequences. Indeed, while TSP1 expression in CRC tumor stroma was shown to inhibit angiogenesis and tumor growth by activating TGF-β1 [99], another group of researcher demonstrated that the over-expression of TSP1 and TGF-β was responsible for more aggressive forms of glioma [100].

Another key player in the stromal activation of TGF-β is the bone morphogenetic protein 1 (BMP1). Using a mouse model of embryonic fibroblasts, Ge et al. report that BMP-1 directly cleaves LTBP1 at its N- and C-terminal sites, resulting in the release of the LLC from the ECM [72]. Their data also demonstrate that the released latent TGF-β complex is further processed via MMP2-mediated proteolysis of the LAP domain (Figure 3A) [72]. TGF-β can also induce the expression of BMP-1, resulting in positive feedback regulation of TGF-β activity. Of note, deregulation of TGF-β/BMP activity can lead to developmental defects and pathological disease, including cancer. Fibulins are reported to play an important role in the modulation of TGF-β activation. They are a family of seven secreted glycoproteins with homologous epidermal growth factor-like domains and a unique C-terminal structure. Fibulins interact with ECM proteins forming anchoring structures that can regulate cell proliferation and migration [101]. Interestingly, similar to TGF-β, the fibulins possess context-specific pro-tumorigenic and tumor-suppressive properties [102]. Of the different fibulins interacting with TGF-β1 (see also stromal inhibitors) fibulin-2 enhances the release of TGF-β1 from its latent complex in the ECM (Figure 3A) [103]. The stimulatory role of fibulin-2 on TGF-β has been further highlighted in a fibulin-2 null mouse model, where depletion of the protein is associated with a decrease in canonical TGF-β signaling activity [104]. Another line of research based on genomic microarray analysis of neuronal stem cells, has reported that inhibition of fibulin-2 leads to the blockade of TGF-β1 mediated pro-neurogenic effects. Of note, TGF-β can also stimulate the expression of fibulin-2, thus establishing a positive feedback loop [105].

Latent TGF-β can also be activated by other stromal-derived proteinases, such as plasmin and thrombin, with numerous studies demonstrating that these enzymes are involved in its indirect activation via LTBP1 cleavage [106,107]. Cleavage of LTBP1 results in the release of matrix-bound TGF-β from the ECM, where it is subsequently activated by MMPs (Figure 3A). Accordingly, a study examining the relevance of human fibroblast and fibrosarcoma-derived pericellular structures in TGF-β regulation has revealed that these matrices act as a storage compartment for latent TGF-β. The addition of plasmin and thrombin to the cellular structures results in the rapid release of LTBP1 [65]. The latter observations are consistent with a number of other studies that demonstrate that the latent form of TGF-β can also be activated by plasmin-mediated proteolysis in co-cultures of endothelial and smooth muscle cells [106,108,109].

## 4. Stromal Inhibitors of TGF-β in Cancer

One of the most significant consequences of TGF-β release into the tumor microenvironment is a robust fibrotic response, known as a desmoplastic reaction. The desmoplastic stroma is characterized by the overproduction of ECM proteins and enhanced proliferation of cells associated with a myofibroblast phenotype [110]. Many solid tumors display a dense and fibrotic stroma, where CAFs, in response to the cytokines released by cancer cells, contribute to the overproduction of ECM components. This process inevitably leads to an increase in matrix rigidity, myofibroblast contractility [111] and, as previously described in this review, the subsequent release of mature TGF-β from the LLC. Thus, TGF-β activation, myofibroblast contraction and ECM remodeling collectively participate in a positive feedback loop that drives the initiation and progression of tumors. In the present section, we discuss stromal proteins known to suppress TGF-β activity.

### 4.1. Proteoglycans

Proteoglycans are a family of highly glycosylated proteins mainly involved in tissue organization and in the regulation of collagen fibrillogenesis [112]. Proteoglycans and in particular small leucine-rich proteoglycan (SLRP) family members, such as decorin, biglycan, asporin, lumican and fibromodulin, exert an inhibitory influence on the TGF-β signaling pathways. The mechanism, however, leading to TGF-β signaling inhibition is variable, largely consisting of SLRP protein binding to: (i) soluble TGF-β; (ii) TGF-β type I and/or type II receptors; or (iii) latent TGF-β, consequently impairing ligand-receptor interactions.

**Figure 4:**
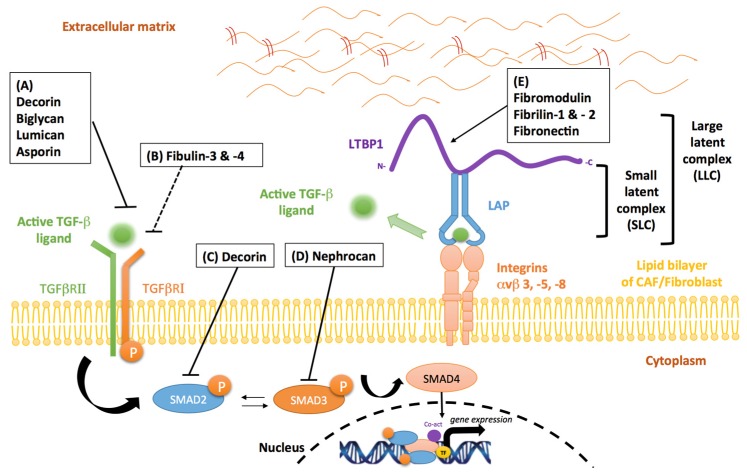
Stromal inhibitors of TGF-β in the tumor microenvironment. (**A**) The SLRPs, decorin, biglycan, lumican and asporin, bind with high affinity to TGF-β, preventing the biologically-active protein from binding to its cognate receptor. (**B**) Fibulin-3 and -4indirectly inhibit TGF-β activity by interacting with TGF-β RI, leading to a decrease in TGFβRI/TGFβRII complex formation. (**C**) Decorin can indirectly disrupt TGF-β activity by negative regulation of SMAD2 phosphorylation. (**D**) Nephrocan indirectly regulates TGF-β activity by inhibiting canonical SMAD3 signaling. (**E**) Fibromodulin and fibrilin-1 and -2 bind to the TGF-β LLC, preventing its release from the ECM. Fibronectin mediates the regulation of LTBP1 in the ECM, thereby suppressing TGF-β1 bioavailability. Solid arrows indicate activation and/or enzymatic activity while blunted arrows indicate inhibitory activity (solid—direct; interrupted—indirect activity).

Decorin, a well-known member of the SLRP family, inhibits TGF-β signaling activity by binding with high affinity to all TGF-β isoforms (Figure 4A). Data also reveal that decorin can act as an endogenous tumor suppressor through sustained inhibition of tumor growth and angiogenesis [113]. More importantly, the loss of stromal decorin predicts poor prognosis in metastatic breast cancer and is associated with a higher incidence of progressive disease [114]. As demonstrated in a murine model of liver fibrosis, decorin null mice display increased expression of both TGF-β1 and its early inducible response gene, TIEG [115]. This loss of decorin expression leads to enhanced accumulation of α-SMA-positive cells and high levels of ERK1/2 and SMAD3 activity [115]. In human mesangial cells, other investigations have demonstrated that decorin disrupts TGF-β/SMAD signaling events (Figure 4C), through a mechanism involving the mobilization of Ca^2+^/calmodium-dependent kinase II and the phosphorylation of SMAD2 at serine-240, a negative regulatory site [116]. In addition to these observations, decorin has also been reported to induce the formation and nuclear translocation of SMAD2/SMAD4 hetero-oligomeric complexes [116]. Therefore, an important aspect controlling decorin-mediated regulation of TGF-β canonical signaling is the nuclear sequestration of cytoplasmic co-SMAD4, rendering it unavailable for TGF-β receptor-initiated SMAD signaling. In malignant glioma cells, decorin contributes to reduced TGF-β pathway activity by preventing the synthesis and release of TGF-β1 and TGF-β2 [117,118]. These events promote tumor regression and prolong survival in experimental murine models [117,118]. Additionally, decorin-mediated inhibition of TGF-β1 activity has been shown to regulate the inflammatory profile of immune cells, such as macrophages [119]. In this cellular subset, suppression of TGF-β1 activity by decorin is associated with reduced levels of miR-21, a post-transcriptional repressor of PDCD4, a pro-inflammatory molecule. The increase in PDCD4 leads to a lower production of the anti-inflammatory cytokine, IL-10, and the ensuing establishment of a pro-inflammatory environment [119].

Biglycan, a small interstitial proteoglycan structurally-related to decorin, is considered a potent modulator of cytokine function due to its ability to bind TGF-β (Figure 4A) [120], TNFα, BMP2, BMP4, BMP6 and WISP1 [121]. In vitro studies conducted in the context of lung fibrosis, have shown that biglycan and decorin can suppress TGF-β activity in a dose dependent manner [122]. However, while the overexpression of decorin is sufficient in reducing TGF-β-induced fibrosis, biglycan does not exert a similar effect. A plausible hypothesis could be that these differences are associated with the distinct distribution of the two SRLPs within tissue compartments [122]. 

Fibromodulin, another SLRP that shares extensive sequence homology with decorin and biglycan, is known for its role in collagen fibril structural organization [123]. Interestingly, several studies have reported that the expression of fibromodulin is significantly reduced in metastatic cancers compared to primary cancers [124,125,126]. In human fibroblasts, fibromodulin expression suppresses nuclear factor-kappa b (NF-κb) signaling through a series of events that results in their diminished survival [127]. Moreover, fibromodulin-mediated regulation of TGF-β occurs through its interaction with latent TGF-β (Figure 4E). Experimental evidence has demonstrated that fibromodulin binds to LLC of TGF-β, preventing its release from the ECM [120,128]. Importantly, the direct influence of fibromodulin on TGF-β bioavailability has recently been uncovered in a fibromodulin-null mouse model of skin wound repair [129]. The absence of fibromodulin in this model was associated with an increase in TGF-β3 bioavailability in cells of stromal origin, as well as a corresponding increase in levels of its cognate receptors, TGFβRI and TGFβRII. However, a recent study by Adini et al. has demonstrated that fibromodulin exerts a stimulatory influence on TGF-β1 expression and secretion in endothelial cells in angiogenesis-dependent disease such as macular degeneration [130]. In their study, endothelial cells incubated with fibromodulin displayed enhanced levels of phosphorylated SMAD1/5, in addition to an increase in migratory and angiogenic capacity. The authors further highlight that fibromodulin-induced secretion of TGF-β1 can co-activate TGFβRII, stimulating canonical SMAD transcriptional complexes [130].Given that emerging evidence has demonstrated the dual function of fibromodulin in TGF-β modulation, further investigation is required to fully determine its pertinent function in different cellular subsets.

Another SLRP known to modulate TGF-β1 activity is asporin. Asporin structurally differs from other SLRP family members due to the presence of an aspartic acid repeat in its N-terminal region and the absence of carbohydrates moieties [131]. Aberrant expression of stromal-derived asporin has been reported to contribute to tumor promoting effects in several human cancers [131,132]. However, our group has been the first to demonstrate a tumor suppressive role for asporin in breast cancer [131]. In our study, we have shown that CAF-secreted asporin leads to the inhibition of TGF-β-induced SMAD2 phosphorylation and the reversal of an EMT phenotype in breast cancer cells. Collectively, our results suggest that asporin directly binds TGF-β1 (Figure 4A), rather than acting as competitive inhibitor of its cognate receptor, TGFβRII. In support of our conclusions, a previous report has established that the interaction between asporin and TGF-β1 occurs between residue His^159^ and Asn^205^ of the asporin amino acid sequence [133]. Interestingly, the expression of asporin positively correlates with active levels of TGF-β1, indicating the existence of a negative feedback loop that can lead to better regulation of TGF-β1 activity [131]. This loop can be disrupted in triple negative breast cancer, where overexpression of IL-1β results in the effective inhibition of CAF-induced asporin expression [131].

Lumican is a TGF-β modulator known to be overexpressed in several solid tumors, however its exact role in tumorigenesis remains poorly understood. Clinical evidence suggests that lumican expression in the stromal compartment of pancreatic and breast malignancies is associated with lower patient survival and a higher incidence of metastatic disease [134,135]. On the other hand, pre-clinical evidence has demonstrated that lumican reduces the proliferative capacity and adhesive properties of osteosarcoma cells in the ECM [136]. Mechanistically this inhibitory activity occurs via high affinity binding of lumican to TGF-β2 (Figure 4A) and the negative regulation of downstream targets such as SMAD2, integrin β1 and FAK [136,137]. Interestingly, down-regulation of lumican mRNA levels can abrogate the latter effects, further indicating that lumican influences the bioavailability of TGF-β2 [136]. 

Nephrocan, a member of a new class of SLRPs, contains the canonical cysteine cluster at the N-terminal region and the 17 leucine-rich repeat (LRR) motif. However, nephrocan differs from other SLRP members, due to the presence of four cysteine residues in the *C*-terminal flanking domain, five potential *N*-glycosylation sites, and a polyacidic amino acid tail at the *C*- terminus. Following the general trend of the other SLRPs, nephrocan has also been reported to function as an endogenous inhibitor of canonical TGF-β signaling, exerting its tumor suppressive function through negative regulation of phosphorylated SMAD3 (Figure 4D) [138]. The entire sequence of events behind nephrocan-mediated inhibition of TGF-β biological activity is, however, very poorly understood and thus requires further exploration. 

### 4.2. Fibrillins

The fibrillins are evolutionally considered as the main structural proteins of microfibrils [139]. These proteins have been described to regulate the bioavailability of local TGF-β during tissue formation and remodeling. The mechanism by which fibrillin-1 inhibits the activation of TGF-β is not well elucidated, but it is thought that it may involve the association of the LLC with fibrillin-1 rich microfibrils (Figure 4E) [140]. Accordingly, fibrillin-1 deficiency, causes the release of elevated amounts of active TGF-β from the ECM, a condition that in Marfan syndrome patients is associated with severe cardiovascular disease, developmental emphysema and skeletal abnormalities [101]. Fibrillin-2 knock-out mice, display reduced bone formation. This phenotype is dependent on the aberrant activation of latent TGF-β via its interaction with the LLC (Figure 4E), and the consequent blunting of OSTERIX expression, the transcriptional regulator of osteoblast maturation, and collagen I, the structural template for bone mineralization [102]. In addition, fibrillin-1 and -2 can directly bind the propeptides of other TGF-β superfamily members, such as BMP2, BMP4, BMP7, BMP10, and GDF8 [104]. The fibrillin-1 promoter has been reported to be hypermethylated in colorectal cancer cell lines [141], colorectal cancer patient samples [142] and endothelial tumor cells [143]. Consequently, the *FBN1* gene can be considered as a potential tumor suppressor and its down-regulation could play a role in tumor angiogenesis.

### 4.3. Fibulins

Fibulin-3, expressed and secreted by both cancer cells and fibroblasts, has recently been reported to be down-regulated in several tumor types [144,145]. Fibulin-3 inhibits the TGF-β canonical signaling pathway essentially by interacting with TGF-β RI (Figure 4B), thus leading to a decrease in TGFβRI/TGFβRII complex formation [146]. As demonstrated in an in vitro model of breast cancer, fibulin-3 overexpression limits TGF-β-induced EMT, cell migration, invasion and endothelial cells permeability [146].In addition, aberrant promoter methylation and the resultant loss of fibulin-3 gene expression, is associated with a higher risk of metastasis and tumor progression in breast cancer [146], CRC [147], lung cancer [148] and hepatocellular carcinoma [149]. Similar to Fibulin-3, Fibulin-4 has also been reported to suppress canonical TGF-β signaling (Figure 4B). Experimental evidence has demonstrated that patients with a cardiovascular pathology harboring a recessive Fibulin-4 mutation, display increased phosphorylation of SMAD2/3 and enhanced CTGF expression in their tissue samples [150]. Additional data from the same study also establishes that an increased level of phosphorylated SMAD2 in patient-derived skin fibroblasts is associated with the mutant fibulin-4 cohort [150]. These observations are in agreement with another pre-clinical investigation demonstrating conditional knockout of fibulin-4 enhances TGF-β signaling activity [151]. However, following the observation that fibulin-4 deficiency results in marked upregulation of ERK1/2 phosphorylation, the investigators have suggested that this protein may also impair non-canonical TGF-β signaling activity [151]. While the aforementioned findings may have a relevance in a cancerous setting, the body of evidence linking the fibulins to tumor development is yet to be fully elucidated.

### 4.4. Fibronectin

Fibronectin is a glycoprotein involved in tissue repair and ECM regulation [152,153]. Similar to many members of the integrin receptor family, fibronectin also plays a critical role in LTBP1 deposition in the ECM of osteoblasts and fibroblasts [152]. Moreover, Dallas et al. have demonstrated that fibronectin is incorporated into the ECM prior to LTBP1 deposition, thus creating a temporary scaffold template that gradually depletes during the maturation of fibrillar networks (Figure 4E) [154]. As previously described in this review, LTBP1 is a critical part of the TGF-β1 latent complex, therefore fibronectin-mediated regulation of LTBP1 in the ECM directly diminishes TGF-β1 bioavailability. However, other data suggests that TGF-β1 can also induce the expression of fibronectin [155] and its receptor, integrin α5β1 [156] in endothelial cells, creating a key positive-feedback loop that regulates ECM deposition and turnover. 

## 5. Clinical Outlook: Targeting Stromal Modulators of TGF-β in Cancer

Considering its multi-modal role in tumor progression, targeting the TGF-β signaling axis represents a promising therapeutic prospect in cancer therapy. Several classes of TGF-β pathway inhibitors have been evaluated in the preclinical setting including TGF-β neutralizing monoclonal antibodies, anti-TGFβ receptor monoclonal antibodies, anti-sense oligonucleotides and small molecule kinase inhibitors. Importantly, some of these agents are currently under various clinical phases of development, demonstrating a good safety profile but modest efficacy in a wide variety of human cancers. The anti-tumor activity and clinical impact of these pharmacological agents has been extensively reviewed by a number of investigators [157,158,159,160]. The lack of significant therapeutic outcomes indicates that we still need to understand more about the complex and intricate mechanisms involved in TGF-β activity. As described previously, the bioavailability of active TGF-β depends significantly on the activity of distinct TGF-β modulators located in the ECM. The regulation of stromal proteins that directly or indirectly alter TGF-β signal transduction cascades might be exploited as a potential therapeutic strategy in targeted cancer treatment. For example, adenovirus mediated decorin gene transfer (Ad-DCN), has been reported to significantly reduce growth in tumor xenografts. (Ad)-mediated transfer and expression of human decorin cDNA induced apoptosis in vivo via overexpression of p21, a potent inhibitor of cyclin-dependent kinases, and Caspase-8. Moreover, data has revealed that the effect of decorin is specific for tumor cells, as neither apoptosis nor growth inhibition was observed in non-cancerous cells [161]. Accordingly, a clinical overview of various anti-cancer agents designed to either suppress or stimulate some stromal activators and inhibitors of TGF-β, is shown in Table 1. 

### 5.1. MMPs

One of the most extensively studied drug targets are the MMPs. TGF-β and MMPs are mutually regulated in normal and cancer tissues. As previously reported in this review, MMPs activate latent TGF-β which in turn, up-regulates MMPs production in both cancer and stromal cells. TGF-β and MMPs, both contribute to the progression and metastatic potential of tumors. Thus, the inhibition of the amplification loop operated between the TGF-β and MMP system in tumor cells could impair cancer dissemination, proliferation and survival. In addition, the validity of MMPs as therapeutic targets has been investigated in several cancer types. MMP inhibitors such as tanomastat, have demonstrated considerable preclinical efficacy and are well tolerated in cancer patients [166]. However, phase III assessment of these inhibitors has demonstrated a lack of survival benefitcompared to standard chemotherapy [175]. A more promising example is doxycycline, an FDA-approved antibiotic, also known to inhibit MMP secretion [176]. Treatment with doxycycline is associated with reduced tumor growth and metastastic spreading in experimental models of breast cancer [169] and pancreatic cancer [177]. Moreover, doxycycline is non-toxic to normal cells, has anti-inflammatory properties and is known to cross the blood-brain barrier, pointing to its therapeutic value in treating brain malignancies [178]. The anti-tumor efficacy of doxycycline is currently being tested in primary cancers (NCT02874430) and metastatic breast cancer (NCT01847976). A key future step is seen in the development of combined therapy that specifically targets the TGF-β-MMP axis. 

### 5.2. Integrins

The integrins, are another class of TGF-β modulators that have been identified as attractive drug targets [179]. The active cross-talk between TGF-β and integrins is triggered in tumors in response to several stimuli including hypoxia, oxidative stress or therapy, and it promotes tumor survival. In a pre-clinical model of non-small cell lung cancer (NSCLC),the concomitant inhibition of TGF-β1 and integrin β3 silencing, resulted in decreased lymph node metastasis. The data provided in the study support the hypothesis that combined targeted therapy toward TGF-β and β3 integrin could be a promising approach to attenuate metastatic lung cancer [180]. Preclinical and clinical studies with various integrin antagonists have demonstrated their effectiveness in blocking tumor progression.Integrin antagonists, currently in clinical trials, include monoclonal antibodies and RGD peptide mimetics. Etaracizumab, a monoclonal antibody targeting integrin αvβ3, showed a good efficacy in preclinical studies and was one of the first drug targeting integrins to enter into clinical trials [179]. However, while etaracizumab demonstrated a favourable toxicity profile in initial clinical studies, it was discontinued after phase II evaluation following negative outcome data. Likewise, intetumumab, a dual-specific monoclonal antibody targeting integrin αvβ3 and αvβ5 has also shown disappointing clinical results. Clinical investigation in melanoma patients demonstrated that intetumumabmonotherapy or its combination withstandard therapy did not improve overall survival compared to placebo [181]. Interestingly, however, cilengitide, a cyclic pentapeptide designed to selectively inhibit integrin αvβ3 and αvβ5, has demonstrated in phase II clinical evaluation, robust anti-tumor activity when combined with systemic therapy in the treatment of recurrent glioma [182]. The clinical activity of cilengitide was unexpectedly less promising in phase III evaluation. In this study, the cilengitide combined with systemic therapy did not improve patient outcome, indicating that it will not undergo further clinical development [172]. Alternatively, the integrin signalling pathway can be suppressed with the use of Focal Adhesion Kinases (FAK) small molecule inhibitors such as VS-6062 and GSK2256098. The efficacy of these inhibitors has been evaluated in phase I clinical trials initiated for several solid tumors, including head and neck cancer, prostate cancer, pancreatic cancer and ovarian cancer [183]. 

### 5.3. Fibronectin

Fibronectin has also been examined as a candidate target in the treatment of malignant disease. However, unlike the previously discussed targets, fibronectin has been developed primarily for antibody-based drug conjugates. Indeed, the abundance, accessibility and stability of certain isoforms of fibronectin suggests it bears excellent properties for the intratumoral delivery of therapeutic antibodies [184]. In cancer and other angiogenesis-related disease, the majority of clinical studies have been performed using the ScFv (L19), an antibody against a fragment of fibronectin. To date, more than 30 L19-based biopharmaceuticals have been tested in preclinical trials. For example, Santimaria et al. have shown that radiolabelled L19 is accumulated in tumor lesions in aggressive types of lung cancer and colorectal cancer [185]. Furthermore, other investigators have demonstrated decreased tumor growth in mice grafted with subcutaneous tumors after intravenous injection of L19 [186]. Interestingly, the L19 antibody has also been fused with a large number of chemical derivatives and fusion proteins such as cytokines and chemokines. In preclinical studies, the fusion of L19 with IL-2 has shown potent tumor-targeting effects and therapeutic efficacy in orthotopic models of hepatocellular carcinoma and pancreatic cancer [187]. The delivery of TNFα to tumor blood vessels has also been investigated by using a fusion protein composed of mouse TNF-α and a high-affinity antibody fragment targeted to the extradomain B of fibronectin (L19mTNF-α) [188]. Radiolabeled L19mTNFα selectively targeted tumor neovasculature and demonstrated significant anti-tumoractivity. Interestingly, the safety and efficacy of L19-TNFα combined with L19-IL2 has been investigated in a phase II clinical study for patients with metastatic melanoma [189]. A single intratumoral administration of the L19-TNFα /L19-IL2 mixture was sufficient to eliminate the tumor, while neither of these agents alone was effective [189]. Finally, the recombinant human fibronectin fragment (FN-CH296, RetroNectin), designedto enhance T-cell therapy in patients with advanced cancers has shown a good safety profile and a high level of efficacy in phase I clinical evaluation [190].

## 6. Conclusions

The importance of TGF-β as a regulator of host-tumor interactions during the initiation and progression of human cancers is well documented. Indeed, the tumor stroma is an important element in cancer development and a large proportion of the cells that mediate stromal-epithelial interactions are of fibroblastic origin. It cannot be excluded, however, that TGF-β- induced bi-directional signaling between epithelial cells and other cell types within the stromal microenvironment, such as endothelial cells and inflammatory cells, may also play an important role in tumor formation. This, however, has been far less investigated.

The present review has explored the role of stromal modulators of TGF-β in cancer. These stromal modulators, however, need not only be limited to cancer, but can also play a role in a host of other pathologies and degenerative disease. The MMPs, integrins, ROS, and other stromal-derived molecules activate TGF-β by assessing the latent cytokine, generating the mature protein and thereby regulating its bioavailability. In addition, under normal physiological conditions a homeostatic balance exists between active levels of TGF-β and its sequestered latent form. However, in pathological conditions such as cancer and inflammation, the integrity of the ECM is lost thus enabling stromal cells such as fibroblasts and CAFs to assess the defective ECM and induce increased expression of activators of latent TGF-β, as part of the process of repair [29]. This shift in balance in the physiological and pathological setting, broadly impacts the bioavailability of TGF-β; its apparent paradoxical effects on malignant cells and their biological processes. On the other hand, the mechanism underlying negative control of TGF-β by inhibitors such as the proteoglycans, fibrillins, fibulins and fibronectin, is more diverse compared to its positive control and activation. The interaction of TGF-β inhibitors and the cytokine are not only limited to their interaction with its sequestered latent form, but also with their inhibition of the activity of downstream signaling protein such as SMAD2 and SMAD3. Moreover, the mature active form of TGF-β can also induce the expression of some of these stromal inhibitors such as fibronectin [155] and asporin [131], further highlighting the paradox of TGF-β and the complexity of its regulation in the tumor microenvironment. Taken together, additional work is needed to better classify these stromal modulators of TGF-β and their impact on malignant disease. Further elucidation will thus depend on further identification of the underlying molecular mechanisms associated with these modulators of TGF-β, through profiling and OMICS approaches. This will ultimately enable greater understanding of the tumor microenvironment and its fundamental process, paving the way for a new era of personalized medicine. 

## Figures and Tables

**Table 1:** Clinical development of agents targeting TGF-β modulators.

Name	Class	Target	Developmental Stage
*Matrix Metalloproteinases*			
Batimastat [162]	Peptidomimetic	Broad spectrum	Cancelled in phase III
Marimastat (BB-2516) [163]	Peptidomimetic	Broad spectrum	Cancelled in phase III
CGS-27023A (MMI270) [164]	Small molecule	MMP-2, 8, 9	Cancelled in phase I
Prinomastat (AG3340) [165]	Small molecule	MMP-2, 3, 9, 14	Cancelled in phase III
Tanomastat (BAY-129566) [166]	Small molecule	MMP-2, 3, 9, 13	Cancelled in phase III
BMS-275291 (D2163) [167]	Peptidomimetic	MMP-1, 2, 9	Cancelled in phase III
Metastat (COL-3) [168]	Tetracycline derivative	Broad spectrum	Phase I/II
Doxycycline [169]	Tetracycline derivative	N/A	Approved/ongoing
*Integrins*			
Vitaxin/etaracizumab [170]	Monoclonal antibody	β7	Cancelled in phase II
Intetumumab (CTNO 95) [171]	Monoclonal antibody	αvβ3, αvβ5	Phase I/II
Cilengitide [172]	Cyclic RGD peptide	αvβ3, αvβ5	Cancelled in phase III
PF-00562271 (VS-6062) [173]	Small molecule	FAK	Phase I
GSK2256098 (NCT00996671)	Small molecule	FAK	Phase I
VS-4718 (PND-1186) [174]	Small molecule	N/A	Phase II
PF-04554878 (VS-6063) (NCT01951690)	Small molecule	FAK, PYK2	Phase II
*Fibronectin*			
L-19 (NCT02076633)	Monoclonal antibody	ED-B domain of fibronectin	Phase II
